# Serum Bilirubin Levels and Disease Severity in Patients with Pneumoconiosis

**DOI:** 10.1155/2023/5642040

**Published:** 2023-03-14

**Authors:** You-Fan Peng, Jun-Hua Deng, Xiao-Ying Huang, Qing-Song Zhang

**Affiliations:** Department of Respiratory and Critical Care Medicine, The Affiliated Hospital of Youjiang Medical University for Nationalities, Baise, China

## Abstract

**Aim:**

To investigate the association between serum bilirubin and disease severity in patients with pneumoconiosis.

**Methods:**

The study comprised 45 patients with pneumoconiosis retrospectively; all pneumoconiosis patients were classified into I, II, and III stage according to the radiological severity.

**Results:**

Serum direct bilirubin levels were significantly lower in III stage pneumoconiosis patients than those in I/II stage (*p* = 0.012) but not serum indirect bilirubin. Serum direct bilirubin was negatively correlated with radiological severity in patients with pneumoconiosis (*r* = −0.320; *p* = 0.032); by multiple linear-regression analysis, we observed that serum direct bilirubin levels had independent association with radiological severity in patients with pneumoconiosis (beta = −0.459; *p* = 0.005).

**Conclusions:**

Serum direct bilirubin levels are negatively associated with disease severity in patients with pneumoconiosis.

## 1. Introduction

Pneumoconiosis is a group of heterogeneous occupational interstitial lung diseases caused by long-term accumulation of dust in the lungs [1]. Although many protective measures are taken to confront dust inhalation in workers, however, pneumoconiosis is still prevalent all around the world, and its morbidity has remained at relatively high levels, which seriously threatens global public health [2, 3]. The timely diagnosis and evaluation of pneumoconiosis have close association with the treatment and prognosis in patients with pneumoconiosis; clinically, screening of pneumoconiosis mainly relies on the exposure history of dust and imaging examination [2]. However, few serum biomarkers have been used to evaluate the disease status in patients with pneumoconiosis.

Serum bilirubin, an end metabolic product of heme catabolism, consists of two forms: direct bilirubin and indirect bilirubin [4]. Serum bilirubin has been used as a traditional marker for liver disease in clinical practice [5]. In recent years, serum bilirubin has been reported to be associated with inflammatory diseases, such as metabolic syndrome, atherosclerosis, and hypertension [6]. In fact, it is generally believed that bilirubin possesses multiple biological activities including antioxidant and anti-inflammatory action [7]. Besides, bilirubin also has cytoprotective properties against oxidative stress [8]. Noteworthy, bilirubin can attenuate pulmonary inflammation by its anti-inflammatory and antioxidant properties in the rat model of smoke-induced emphysema [9], and antioxidant action of bilirubin can ameliorate bleomycin-induced pulmonary fibrosis by inhibiting lung inflammation [10]. Pulmonary inflammation and oxidative stress have been accepted to be involved with inhalation of dust in the lungs [11, 12]. Therefore, we examined the relationship between serum bilirubin and disease severity in patients with pneumoconiosis.

## 2. Materials and Methods

Our study included 45 patients with pneumoconiosis retrospectively; all pneumoconiosis patients were diagnosed according to the radiological features and history of occupational exposure. Our study excluded patients with the following conditions: coronary heart disease, hepatic insufficiency, renal insufficiency, hemolytic disease, gallstone disease, chronic obstructive pulmonary disease, asthma, bronchiectasis, lung abscess, autoimmune disease, and malignant tumors. We used radiological severity to classify pneumoconiosis patients as I, II, and III stage, which is similar compared with the classification system of the International Labor Organization criteria [13–15]. The study was approved by the Ethics Committee of The Affiliated Hospital of Youjiang Medical University for Nationalities and conformed to the Declaration of Helsinki.

### 2.1. Statistical Analysis

Means ± standard deviation or median (interquartile range) was used as descriptive statistics for normal distributed or non-normal continuous variables, respectively. Percentage was used as descriptive statistics for categorical variables. The Chi-square or Fisher's exact test was used to compare the differences of categorical variables. Comparative analyses of two continuous variables were performed with Student's *t*-test or Mann–Whitney *U* test, when appropriate. The bivariate correlation was examined by Pearson or Spearman approach appropriately. Multiple linear-regression analysis was further carried out for identifying the independent determinants. All statistical analyses were performed using SPSS 25.0 software version. A *p* value <0.05 considered as statistically significant.

## 3. Results

### 3.1. Comparison of Serum Bilirubin Levels among Pneumoconiosis Patients with Different Stages

**Figure 1:**
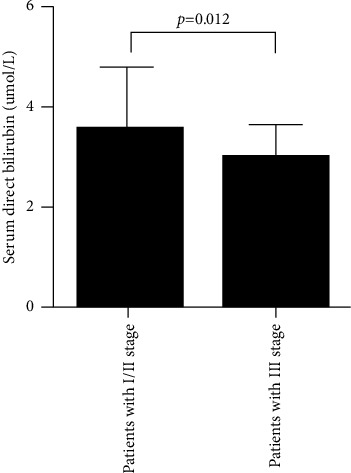
The comparison of serum direct bilirubin between stage I/II pneumoconiosis and stage III pneumoconiosis.

**Figure 2:**
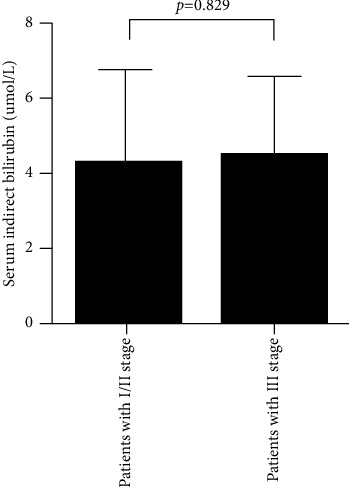
The comparison of serum indirect bilirubin between stage I/II pneumoconiosis and stage III pneumoconiosis.

The characteristics of patients are described in Table 1. Patients with III stage pneumoconiosis are usually regarded as advanced pneumoconiosis clinically; hence, the differences of serum direct bilirubin and serum indirect bilirubin were compared between stage I/II and stage III pneumoconiosis patients; we found that serum direct bilirubin levels were significantly reduced in III stage pneumoconiosis patients compared with those in I/II stage (*p* = 0.012) (Figure 1); serum indirect bilirubin levels have no significant differences between the two groups (Figure 2). There were no significant differences with respect to sex, age, smoking history, pulmonary tuberculosis history, diabetes mellitus, hypertension, total protein, alanine aminotransferase, and aspartate aminotransferase.

### 3.2. The Correlation Analysis between Serum Bilirubin and Disease Severity in Pneumoconiosis Patients

Our study estimated whether serum bilirubin levels were correlated with disease severity in patients with pneumoconiosis; the correlation analysis was performed in patients with pneumoconiosis; the results indicated that serum direct bilirubin had a negative correlation with radiological severity in patients with pneumoconiosis (*r* = −0.320; *p* = 0.032); no correlation between serum indirect bilirubin and radiological severity was observed in patients with pneumoconiosis.

### 3.3. Multiple Linear-Regression Analysis with Serum Direct Bilirubin as Dependent Variable

Multiple linear-regression analysis was used to identify independent determinants associated with serum direct bilirubin in patients with pneumoconiosis. When sex, age, smoking history, alanine aminotransferase, aspartate aminotransferase, and radiological severity were included as independent variables, the multiple linear-regression analysis revealed that serum direct bilirubin levels were independently associated with radiological severity in patients with pneumoconiosis (beta = −0.459; *p* = 0.005), as shown in Table 2.

## 4. Discussion

Over the years, serum bilirubin has been reported to be associated with various diseases. For instance, lower serum bilirubin is associated with disease activity in patients with Crohn's disease [16], and lower serum bilirubin levels are noticed in patients with coronary artery disease [17]. Recently, serum bilirubin has been introduced as a potential biomarker in patients with pneumoconiosis [18]. In our study, serum direct bilirubin levels were lower in patients with pneumoconiosis with stage III than in those with I/II stage, and an association between serum direct bilirubin and radiological severity was observed in patients with pneumoconiosis.

Bilirubin as an endogenous lipid-soluble antioxidant has been highlighted its antioxidant effects [19]. Indeed, serum bilirubin may be a useful parameter to reflect oxidative stress in vivo [20], and bilirubin has much stronger antioxidant activity than many other antioxidants [21]; there is a correlation between plasma bilirubin and oxidative-stress markers in HIV-infected patients [22]. In addition to the effects on the oxidative stress, bilirubin also plays a role in anti-inflammation and contributes to improve tissue injury through its anti-inflammatory mechanism in sepsis [23]; serum bilirubin is correlated with low serum C-reactive protein levels in apparently healthy adults [24]. There is an inverse correlation between serum bilirubin and high sensitive C-reactive protein in patients with metabolic syndrome, lower serum bilirubin levels are involved in enhanced low-grade systemic inflammation [25]. Moreover, the significant inverse correlation between serum bilirubin and inflammatory markers has also been suggested in patients with coronary artery disease [26]. Importantly, bilirubin is helpful for inhibiting lung inflammation due to its excellent antioxidant and anti-inflammatory properties [9, 10]. Thus, given that bilirubin may be consumed due to prolonged inflammation and oxidative stress in pneumoconiosis; lower serum bilirubin levels may be caused due to an overconsumption of bilirubin in patients with pneumoconiosis.

Interestingly, no significant correlation between serum indirect bilirubin and radiological severity was observed among patients with pneumoconiosis. In fact, indirect bilirubin is insoluble in water, and it is tightly bound to albumin [27]; direct bilirubin is weakly bound to albumin and is easily separated from albumin and translates into active form compared with the other bilirubin subtypes when direct bilirubin acts on a molecule or organ to perform its function [28]. Thus, direct bilirubin may carry out its functions of anti-inflammation and antioxidative stress in patients with pneumoconiosis.

Recently, a relationship between serum bilirubin and lung function decline has been demonstrated in patients with chronic obstructive pulmonary disease [29]. Apperley S et al. [30] suggested that serum bilirubin is associated with forced expiratory volume in 1 s in patients with mild chronic obstructive pulmonary disease. These findings suggest that lung function may influence serum bilirubin levels in chronic obstructive pulmonary disease. In our study, to avoid an influence from the decline in lung function on the association between serum bilirubin and radiological severity in patients with pneumoconiosis, our study design excluded chronic obstructive pulmonary disease in patients with pneumoconiosis. In addition, smoking is a significant contributor for reduced lung function [31], so we further adjusted smoking in the multiple linear-regression analysis, thereby avoiding the effects of smoking on present results in patients with pneumoconiosis.

Regarding the limitations of the study. First, the sample size of the current study is small, especially for subgroup analysis of pneumoconiosis patients. Second, our study did not clarify the effects of anti-inflammatory or antioxidant therapy such as glucocorticoid use on serum bilirubin in patients with pneumoconiosis, particularly for inhaled therapy of glucocorticoids. Third, our study did not analyze the association between bilirubin and inflammation or oxidative-stress marker in patients with pneumoconiosis.

## 5. Conclusions

In conclusion, our study demonstrates a negative association between serum direct bilirubin and disease severity in patients with pneumoconiosis. However, further studies are warranted to validate the role of serum bilirubin in patients with pneumoconiosis.

## Figures and Tables

**Table 1:** Clinical and laboratory characteristics of the pneumoconiosis patients.

Variables	
Male, *n* (%)	42 (93.3)
Age (years)	52 ± 9
Smoking history, *n* (%)	24 (53.3)
Pulmonary tuberculosis history, *n* (%)	15 (33.3)
Diabetes mellitus	1 (2.2)
Hypertension	6 (13.3)
Radiological severity	
I/II stage, *n* (%)	21 (46.7)
III stage, *n* (%)	24 (53.3)
Total protein (g/L)	66.5 ± 5.5
Alanine aminotransferase (U/L)	17.0 (13.2–21.6)
Aspartate aminotransferase (U/L)	20.8 (18.0–24.5)
Serum direct bilirubin (*μ*mol/L)	3.2 ± 1.1
Serum indirect bilirubin (*μ*mol/L)	3.9 (3.0–6.3)

**Table 2:** The association between serum direct bilirubin and radiological severity according to the multiple linear-regression analysis.

	Unstandardized coefficients		Standardized coefficients	*t*	*p* value
	B	Standard error	Beta		
Sex	0.123	0.661	0.028	0.186	0.853
Age	0.015	0.019	0.126	0.784	0.438
Smoking history	0.135	0.343	0.063	0.393	0.696
Alanine aminotransferase	−0.028	0.026	−0.175	−1.052	0.300
Aspartate aminotransferase	0.042	0.041	0.168	1.009	0.319
Radiological severity (I/II vs. III stages)	−0.992	0.335	−0.459	−2.957	0.005

## Data Availability

The data used to support the findings of this study can be obtained from the corresponding author upon reasonable request.
